# Non-Invasive Blood Pressure Estimation from ECG Using Machine Learning Techniques

**DOI:** 10.3390/s18041160

**Published:** 2018-04-11

**Authors:** Monika Simjanoska, Martin Gjoreski, Matjaž Gams, Ana Madevska Bogdanova

**Affiliations:** 1 Faculty of Computer Science and Engineering, Ss. Cyril and Methodius University, Rugjer Boshkovikj 16, 1000 Skopje, Macedonia; ana.madevska.bogdanova@finki.ukim.mk; 2 Department of Intelligent Systems, Jožef Stefan Institute, Jožef Stefan International Postgraduate School, Jamova cesta 39, 1000 Ljubljana, Slovenia; martin.gjoreski@ijs.si (M.G.); matjaz.gams@ijs.si (M.G.)

**Keywords:** blood pressure, ECG, machine learning, complexity analysis, classification, regression, stacking

## Abstract

Background: Blood pressure (BP) measurements have been used widely in clinical and private environments. Recently, the use of ECG monitors has proliferated; however, they are not enabled with BP estimation. We have developed a method for BP estimation using only electrocardiogram (ECG) signals. Methods: Raw ECG data are filtered and segmented, and, following this, a complexity analysis is performed for feature extraction. Then, a machine-learning method is applied, combining a stacking-based classification module and a regression module for building systolic BP (SBP), diastolic BP (DBP), and mean arterial pressure (MAP) predictive models. In addition, the method allows a probability distribution-based calibration to adapt the models to a particular user. Results: Using ECG recordings from 51 different subjects, 3129 30-s ECG segments are constructed, and seven features are extracted. Using a train-validation-test evaluation, the method achieves a mean absolute error (MAE) of 8.64 mmHg for SBP, 18.20 mmHg for DBP, and 13.52 mmHg for the MAP prediction. When models are calibrated, the MAE decreases to 7.72 mmHg for SBP, 9.45 mmHg for DBP and 8.13 mmHg for MAP. Conclusion: The experimental results indicate that, when a probability distribution-based calibration is used, the proposed method can achieve results close to those of a certified medical device for BP estimation.

## 1. Introduction

In the European Union (EU), 1.7 million persons younger than 75 years old died in 2013. Nearly 34% of those deaths could have been avoided if the patients had been provided with better healthcare [1]. Similarly, in 2010, the World Health Organization (WHO) accredited 63% of global deaths to non-communicable diseases that are largely preventable [2]. The published statistics clearly showcase that there is significant room for improvement in the field of personal healthcare in the EU and worldwide. Around half of avoidable deaths are related to heart attacks and strokes. Blood pressure (BP) increases, among other factors, heighten the risk of cardiovascular diseases, strokes, renal failure [3], and arterial stiffness [4]. Thus, hypertension thresholds need to be established for appropriate and timely treatments [5]. A typical biomedical signal processing system encompasses the biological system of interest, the sensors used to capture the activity of the biomedical system, and the methodology developed to analyze the signals and extract the desired information from the activity under scrutiny. In our study, the biological system being analyzed is the heart system, whose electrical activity is illustrated through ECG signals. There are two phases for managing blood flow: a diastole phase known as the filling phase and a systole phase known as the pumping phase. Blood pressure is defined as the force of the blood pushing against the walls of the arteries as the heart pumps blood [6] and is measured in millimeters of mercury (mmHg). A normal heart rate is considered to be 70 beats per minute [7]. The maximum pressure during one heart beat is the SBP and the minimum pressure in between two heart beats is the DBP. Recent technological advances have brought wearable bio-sensors (e.g., ECG sensors, sweating-rate sensors, respiration-rate body sensors, etc.) into everyday life. Wearable bio-sensors provide an opportunity for real-time monitoring of human vital signs, thus enabling the possibility for preventive, timely notification and real-time diagnosis [8]. Unlike commonly-used BP sensors, which demand a specific measurement procedure, modern wearable bio-sensors monitor vital signals on line and all day long, presenting no additional burden other than wearing the device. Many of the devices, even the low-cost ones, achieve reasonable results in real-life circumstances. Some of the systems developed for the purpose of non-invasive BP monitoring are: the Superficial Temporal Artery Tonometry-based device [9], the PPG optical sensor [10], ARTSENS (ARTerial Stiffness Evaluation for Non-invasive Screening) for brachial arterial pressure [11], an electronic system based on the oscillometric method [12], a BP estimation device based on the principle of volume compensation [13], a Modulated Magnetic Signature of Blood mechanism [14], and portable equipment that includes a cuff-based BP sensing system [15]. However, all of these devices exist as standalone devices that are specialized only for BP measurements, and exclude the other vital signs. Additionally, some of them do not achieve the desired laboratory results when used in real life, in particular for specific groups of users.

Regarding the research on BP estimation, most studies use a combination of electrocardiogram (ECG) and photoplethysmogram (PPG) sensors [16,17,18], making the problem even more complex and equipment necessary. The common techniques used for obtaining measurements on BP mainly rely on Pulse Wave Velocity (PWV), Pulse Arrival Time (PAT), and Pulse Transit Time (PTT) [19,20,21,22], all of which require an accurate PPG measurement, which is not simple to get unobtrusively yet, and no clear proof on the PPG measurement’s relation to the BP has been provided [23,24,25]. Considering the methods that use ECG sensors, Chan et al. [26], and Ahmad et al. [27] have presented studies on this relationship; however, both methods used an additional sensor besides the ECG sensor, i.e., the PPG sensor. The ECG-BP relationship has previously been discussed and tested in few studies [28,29]; however, the results confirm no strong relationship between hypertension occurrence and morphological changes in ECG. For this reason, we approach the problem from a different perspective in that our approach does not rely on ECG morphological changes.

In this paper, we present a method that uses the ECG signal as the only source of information for estimating the maximum value of an arterial pressure tracing (SBP), the minimum value of an arterial pressure tracing (DBP), and the MAP value calculated from the SBP and DBP reference values. This method has the potential to be applied on wearable sensors, enabling unobtrusive on line all day measuring of BP parameters. Since wearable sensors technology has been applied in various situations, from ambulatory and clinical situations [30], to military environments [31], this tool may be an appropriate solution that will decrease the need for various sensors to be attached to the human body. Considering urgent medicine, the four parameters HR, RR, BP, and SPO2 are the essential vital signs to establish the hemostability of an injured person [32]. Up until this point, in real situations, BP can be measured only by a standalone BP device in the vehicles or hospitals. On the other hand, modern telemedicine has allowed for the development of systems that use on-field patch-like ECG bio-sensors attached to a patient’s chest. The method we are proposing can derive BP measurement from the ECG signal only.

The approach is based on a combination of complexity analysis and machine learning to detect bio-system complexity and then use AI-based methods to infer medical relations. The complexity analysis [33] of the ECG signals is used to exclude the morphological features of the ECG signals. The hypothesis that complexity decrease in the case of abnormality was proven empirically for other medical conditions [34,35,36]. Following this hypothesis, we perform a complexity analysis of 3129 ECG signals obtained from 51 subjects of different age groups in either healthy, unhealthy or traumatic conditions. For experimental purposes, the measurements were performed by using three types of commercial bio-sensors and specialized medical equipment for comparison. The acquisition of the reference BP values for the bio-sensor measurements was performed manually by using an intermittent cuff-based method, whereas, for the clinical measurements, a continuous arterial BP monitor was used. The reference BP measurements were used to categorize the ECG signals into three BP classes (Normal, 0; Prehypertension, 1; and Hypertension, 2), which consisted of the following groups [37]: hypotension (HPTN) and normal (N) as Normal class, prehypertension (PHTN) as Prehypertension class, and stage 1 hypertension (S1HTN), stage 2 hypertension (S2HTN), isolated systolic hypertension (ISHTN), and hypertensive crisis (HTNC) as Hypertension class. Having extracted features by using complexity analysis, a stacking Machine-Learning (ML) solution was applied to classify the ECG signals into the appropriate BP category and, consequently, regression models were developed to predict the SBP, DBP and MAP values for the given ECG signal. The rest of the paper is organized as follows. The proposed method and the data used are described in Section 2.2. The experimental results are presented in Section 3, followed by a discussion in Section 4, where we make a direct comparison between our method and related methods for BP estimation. In Section 5, we present the conclusions of the study.

## 2. Materials and Methods

### 2.1. Materials

The data acquisition for this research can be described in two main steps. The first step is data collection, and the second step is data preprocessing.

#### 2.1.1. Data Collection

We use four distinct sources, three of which were obtained from commercial ECG sensors and one from clinical equipment. To measure the reference SBP and DBP values, we used an electronic sphygmomanometer in parallel with using the commercial sensors, except in the clinical case where the BP reference values were obtained from the invasive BP measurements. All the participants involved in the measurements have signed an agreement for their anonymous data to be included in the study. Each dataset is explained as follows:

1. The Cooking hacks 3-lead ECG sensor [38] was used to measure the ECG signals of sixteen participants between the ages of 16 and 72. This sensor has been used previously in studies that included monitoring in pre-hospital and in-hospital environments [39], for related studies as a method development for estimating the pulse transit time and the pulse wave velocity [40], and has also been esteemed as a valid and reliable low-cost wearable sensor in real environments [41]. Given the available literature, we consider this sensor to be reliable for our research as well. All measurements were performed in a sitting position for a duration of up to 60 s at a sampling rate of 125 Hz, and each ECG signal is accompanied by reference values for the SBP and DBP.
2. Another three participants between ages of 25 and 27 were recorded using the 180 eMotion FAROS [42], a 3-lead ECG sensor with a 1000 Hz sampling rate. eMotion FAROS is confirmed to be a medical-grade device for recording ECG [43]. Thus, it has been used in the latest studies investigating sleep disorders [44] and physical activities of preschool children [45]. Every participant was required to keep the sensor attached to his/her chest for at least 4 h during his/her daily activities, measuring the SBP and DBP periodically in times ranging from 30 min to 1 h.
3. The third sensor used was the Zephyr Bioharness [46] single-lead ECG biosensor at a 250 Hz sampling rate. Zephyr Bioharness has been proven to be a reliable and valid multi-variable monitoring device in several recent studies focused on testing the validity and reliability of the module [47,48,49,50]. Fourteen participants were recorded following the same criteria used for the first case, and 11 additional patients of ages 20–73 were recorded by a physician at the General Hospital in Celje, Slovenia. All participants, excluding the 11 patients recorded at the hospital, had no history of heart problems. Regarding the health status of the 11 patients with heart problem histories, they were reported to suffer from obesity, tachycardia, or myocardial infarction. The measurements of the healthy participants were performed by volunteers working in our group and by ourselves, whereas the measurements in the hospital were taken by a physician—an anesthesiologist.
4. The fourth dataset is composed by recordings obtained from the Charis [51] Physionet database [52]. Those measurements were taken by physicians and using hospital equipment, including routinely employed clinical monitors for the multi-channel ECG recordings and an indwelling catheter for the blood pressure measurements, in the surgical intensive care unit of the Robert Wood Johnson Medical Center at Rutgers University. We obtained ECG and arterial BP recordings for seven patients between the ages of 20 and 74 with traumatic brain injuries.

All information is summarized in Table 1.

#### 2.1.2. Data Preprocessing

The data preprocessing was carried out by data segmentation and labeling. To enable ECG signals labeling, the continuous arterial BP recording was divided into intervals of 30 s, and the values were transformed into single SBP and DBP values. For the discrete BP measurements, no preprocessing was needed and their values were immediately matched to the corresponding ECG signal. Table 2 presents the medical rules for ECG signals labeling with respect to SBP and DBP ranges and the corresponding number of instances in each class. We grouped the neighbouring classes to represent three BP states [37]: normal, prehypertension and hypertension. The grouping provided more balanced classes for the ML classification method. The column “Grouped” presents the group to which each label belongs. For example, there are 25 instances with SBP ≦ 90 or DBP ≦ 60 that belong to the category HPTN. These instances were joined with the instances from category “N” to form the first class (class 0 in Table 3).

Finally, Table 3 presents the overall number of instances, i.e., signal samples (multiple signal samples/instances may refer to one participant) per dataset (sensor, labeled from 1 to 4), the number of participants per dataset, and the number of instances per dataset in each blood pressure class, using the grouping in Table 2 and referred to as 0 (normal), 1 (prehypertension) and 2 (hypertension). The number of participants is not related to the overall number of instances, since more measurements have been taken for some participants than others.

### 2.2. Methods

**Figure 1:**
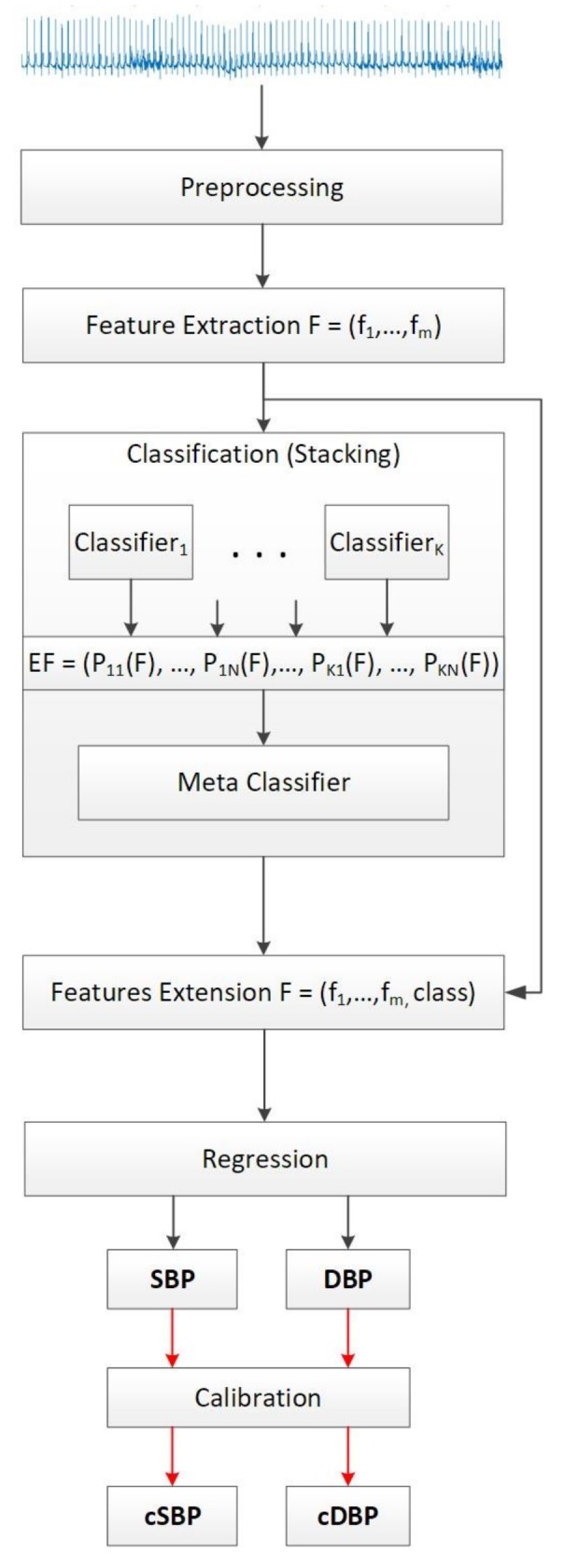
Proposed methodology for blood pressure estimation.

The proposed method is depicted in Figure 1. The raw ECG signals are segmented into 30 s, each accompanied by SBP and DBP values, after which we also compute the MAP values according to Equation (9). Those values pass through the preprocessing method for segmenting the signals, labeling the segments into the appropriate BP class (according to the rules in Table 2), and applying a band-pass filter to preserve only valid ECG information. Following this, the signals are forwarded to the module for complexity analysis and feature extraction. Having computed the complexity metrics as described later in the same section, the feature vectors are inputted to the classification module, which implements a stacking ML approach. The output of the classification module, in combination with the extracted features, is inputted to a regression module, which outputs the BP estimation. The last module is a calibration module that is based on probability distributions of the validation set errors (Section 2.6). The details for each module are presented in the following subsections.

### 2.3. Preprocessing and Feature Extraction Using Complexity Analysis

The accepted range for ECG information is 0.05 up to 100 Hz [7], but an ECG signal downsampled at 50 Hz is considered to preserve all the valid ECG information [53]. Considering lowest frequency of the sensors used is 50 Hz, a band-pass Butterworth filter was applied to the frequencies from 0.3 Hz up to 50 Hz. The threshold of 0.3 Hz was chosen as a reasonable frequency that assures complete baseline removal without deforming the ECG signal [54]. Empirically it has been proven that 30-s segments of ECG signal is enough to create robust estimations in the related field of research [55,56,57], thus we took into account this threshold and trimmed all the signals accordingly. In addition, the 30-s signal length does not transcend the approximate time needed to perform the traditional cuff-based BP measurements.

Traditionally, the ECG signals are processed to extract and analyze the morphological features of the signal [20,23,24,25,58,59,60]. Unlike these approaches, we rely on complexity analysis for representing the information encoded into the ECG signals and model the relation to the SBP, DBP, and MAP values by following a specific ML design. We hypothesize that a normal and healthy biomedical system is highly complex, and once an abnormality occurs, its complexity drops [33,34,35,36,61,62]. Considering related work [63,64,65,66], we selected five metrics as features to model the complexity of the ECG signals: signal mobility, signal complexity, fractal dimension, entropy, and autocorrelation. The features from complexity analysis are invariant to the lead number of the ECG recording. Additionally, we add a feature the age of the participant to the feature vector. Each feature is formally described as follows.

#### 2.3.1. Signal Mobility

Given $x_{i}$, i=1,…,N is the ECG signal of length *N* and $d_{j}=x_{j+1}-x_{j}$ is the first-order variations in the signal, then the first-order factors, $S_{0}$ and $S_{1}$, are calculated as:

(1) $$S_{0}=\sqrt{\frac{\sum_{i=1}^{N}x_{i}^{2}}{N}}$$

(2) $$S_{1}=\sqrt{\frac{\sum_{j=2}^{N-1}d_{j}^{2}}{N-1}}$$

The signal mobility quantitatively measures the level of variation in the signal. It is calculated as a ratio between the factors $S_{1}$ and $S_{0}$:

(3) $$Mobility=\frac{S_{1}}{S_{0}},$$

#### 2.3.2. Signal Complexity

Given the first-order variation of the ECG signal $d_{j}$, j=1,…,N−1, the second-order variation of the signal is presented by $g_{k}=d_{k+1}-d_{k}$. Then, the second-order factor is calculated as:

(4) $$S_{2}=\sqrt{\frac{\sum_{k=3}^{N-2}g_{k}^{2}}{N-2}},$$

Both the signal mobility and signal complexity were computed by using the Hjorth parameters method [67].

#### 2.3.3. Fractal Dimension

The self-similarity of the signal is measured through the fractal dimension. It describes the fundamental patterns hidden in the signal by zooming and comparing different portions. To calculate the fractal dimension, we used the Higuchi algorithm [68] and the parameter settings as described in [69]. The method works with a set of *k* subseries with different resolutions, creating a new time series $X_{k}$, for m=1,…,k:

(5) $$X_{k}^{m}:x\left(m\right),x\left(m+k\right),x\left(m+2k\right),\ldots ,x\left(m+\left\lfloor \frac{N-m}{k}\right\rfloor k\right)$$

The length of the curve $X_{k}^{m}$, l(k) is calculated as:

(6) $$l\left(k\right)=\frac{\left(\sum_{i=1}^{\left\lfloor N-m/k\right\rfloor }|x\left(m+ik\right)-x\left(m+\left(i-1\right)k\right)|\left(N-1\right)\right)}{(\left\lfloor \frac{N-m}{k}\right\rfloor )k}$$

Then, for each *k* in range 1 to $k_{max}$, the average length is calculated as the mean of the *k* lengths l(k) for m=1,…,k. The fractal dimension is the estimation of the slope of the plot ln(l(k)) vs. ln(1/k).

#### 2.3.4. Entropy

The randomness of the signal is expressed through entropy. The decrease of entropy often indicates a disease (an abnormal activity of the biological system measured) [70]. The amount of information is expressed through the concept of probability. Let $p_{i}$ denote the probability of each outcome $x_{i}$ within the ECG signal *X* for i=1,…,N−1. Then, entropy is calculated as:

(7) $$Entropy=\sum_{i=0}^{N-1}p_{i}\log\left(\frac{1}{p_{i}}\right)$$

#### 2.3.5. Autocorrelation

Autocorrelation measures the similarity between the signal and its shifted version. Let τ be the amount of shift, and then the autocorrelation is calculated as:

(8) $$r_{xx}\left(\tau \right)=\int_{-\inf}^{+\inf}x\left(t\right)x\left(t-\tau \right)p_{xx}\left(x\left(t\right),x\left(t-\tau \right)\right)dt,$$

where $p_{xx}\left(x\left(t\right),x\left(t-\tau \right)\right)$ presents the joint probability density function of x(t) and x(t−τ).

#### 2.3.6. Age

The relationship between age and blood flow has been proven by a mathematical model [71] explaining the effect of blood vessel size on blood flow. Actually, age contributes to the changes in the arterial wall that cause the vessels to become stiffer and, thus, the pressure wave velocity increases. Consequently, the reflected pressure waves also move faster back to the heart causing greater systolic pressure to handle the load [71]. Considering this influence, we included the subject’s age in the feature vector. The subject’s age was mapped in five categories, in agreement with the aging process proposed in [72]. The mapping rules and the total number of instances in each age group is given in Table 4.

### 2.4. Classification

The classification is implemented by following a stacking ML design. The stacking module involves a training of seven different algorithms that can model different structures in the data. We used:

1. KNN—to consider inter-instance similarity;
2. J48—to consider the information gain of the features;
3. Naive Bayes—to consider strong independence between the features;
4. SVM—to recognize the most distinguishable feature vectors;
5. Random Forest—to combine multiple models built on varying features set in an ensemble;
6. Bagging—to introduce dataset sub-sampling as a way of reducing the variance of the J48 algorithm; and
7. Boosting—to introduce instance weighting in the dataset for addressing the mis-recognized instances.

Given the feature vector, which was obtained by using the previously described complexity analysis procedure, each classifier produces prediction probabilities [73] for the instance to belong in each of the BP classes defined in Table 2. The probabilities produced, $p_{11}\left(F\right),\ldots ,p_{KN}\left(F\right)$, for each feature vector *F*, of each classifier K=1,…,7 for each BP class, N=1,…,7 are aggregated into new feature vectors that are fed into a single meta-classifier, which in this case is experimentally chosen to be the Random Forest method. The output of the meta-classifier is a new feature that is included in the initial feature vector to be used for the regression analysis (Figure 1).

### 2.5. Regression

A regression is applied to determine the absolute SBP and DBP values, followed by the MAP. Given the SBP and DBP, the MAP value is calculated as:

(9) $$MAP=\frac{SBP+2\times DBP}{3}$$

As explained in the previous section, the initial feature vectors *F* used for developing the classification model are extended by including the original BP class of the training subjects and the output from the classification. As for the testing subjects, the predicted class is included, since it is assumed to be unknown. Having the newly extended feature vectors, $EF=(f_{1},\ldots ,f_{6},c)$, three distinct Random Forest regression models are developed for predicting the SBP, DBP, and MAP values, respectively.

### 2.6. Calibration

Calibration can be a serious issue when using multiple physiological signals [74], but it is also a common problem when developing a BP prediction system, i.e., it usually requires subject-based calibration [75]. We perform calibration by considering the probability distributions of the errors produced by the SBP, DBP, and MAP predictions from the validation set.

## 3. Results

The data were randomly split into three different non-overlapping datasets: 60% of the subjects were included in the training, 10% in the validation and 30% in the testing set. Four different models were built: a classification model that predicts the BP class, which is needed for three different regression models built to predict the real SBP, DBP, and MAP values.

### 3.1. Training Experiments

The preprocessing and the feature extraction phase produced a total of 3129 vectors containing seven features mapped into three BP classes: normal, prehypertension and hypertension, according to the Table 2. Considering the output of the classification model to be a very important as part of the feature vectors upon which the regression models are built, we chose the classification model that performed the best among 100 randomly-chosen train-validation-test sets. The performance of the stacking ML solution used for the classification was evaluated through the kappa statistics that in addition to the accuracy of the classifier also takes into account the possibility of guessing the class by chance as described in Equation (10).

(10) $$\begin{array}{c} Kappa=\frac{success\_rate\_of\_actual\_predictor-success\_rate\_of\_random\_predictor}{1-success\_rate\_of\_random\_predictor} \end{array}$$

After the evaluation of the validation sets in the particular iteration, the number of candidate models was reduced to eight models that obtained kappa statistics of over 0.40. The accuracy, the kappa statistics from the classification, and the errors in mmHg obtained when used in the regression models for each of the validation sets are presented in Table 5.

The choice of appropriate model depends on the performance of the regression models created for each of the eight candidate training sets according to the Kappa statistics, taking into account the errors and the correlation between the real and predicted BP values in the validation set.

Three distinct models for predicting the SBP, DBP, and MAP were created for each of the candidate training sets that showed greatest accuracy when evaluated with the validation set. The original BP classes in the training sets were used to extend the initial feature vectors and prepare the data for regression, as depicted in Figure 1. The regression models were evaluated by using the Mean Absolute Error (MAE) and Root Mean Squared Error (RMSE). MAE is the average error obtained from the absolute differences between the real, $a_{i}$, and the predicted values $p_{i}$, for i=1,…,n, where *n* is the number of instances per subject. MAE weights all the differences equally and is calculated as:

(11) $$MAE=\frac{\sum_{1}^{n}\left|p_{i}-a_{i}\right|}{n}$$

To obtain a higher weight for the large errors, which is important for the BP problem, the differences between the real absolute and the predicted values are first squared, then averaged, and afterwards a square root of the average is taken. The RMSE is calculated according to the following equation:

(12) $$RMSE=\sqrt{\frac{\sum_{1}^{n}{\left|p_{i}-a_{i}\right|}^{2}}{n}}$$

Those results are presented in Table 5 for each SBP, DBP and MAP. The last performance metric that we take into account is the correlation of the real and the predicted BP values in the validation set. Those results are presented in the last column of Table 5.

Given the results, the model with the highest kappa statistic of 0.78 has been chosen as most suitable with achieved accuracy of 85.71%, and errors of 7.86 mmHg, 6.00 mmHg and 11.19 mmHg for the SBP, DBP, and MAP, correspondingly, producing an average correlation of 0.77 between the actual and the predicted values.

### 3.2. Testing Experiments

Table 6 presents the MAE and RMSE evaluation for SBP, DBP, and MAP for each of the 15 subjects (denoted from 1 to 15 in Column 1) included in the testing set. The number of instances per test subject ranges from 1 to 436 (Column 2). The total number of tested instances is 786, producing an overall MAE ± SD (RMSE) in mmHg of 8.64 ± 10.74 (10.97) for the SBP case, 18.20 ± 8.45 (19.34) for the DBP case, and 13.52 ± 8.06 (15.07) for the MAP case. Considering the result for each subject separately, the worst results we saw were from subjects 9, 10 and 12. For those subjects, only one instance is available and we are not able to check the predictions for other instances from the same subject, thus we cannot be assured that the particular measurement obtained is reliable.

**Figure 2:**
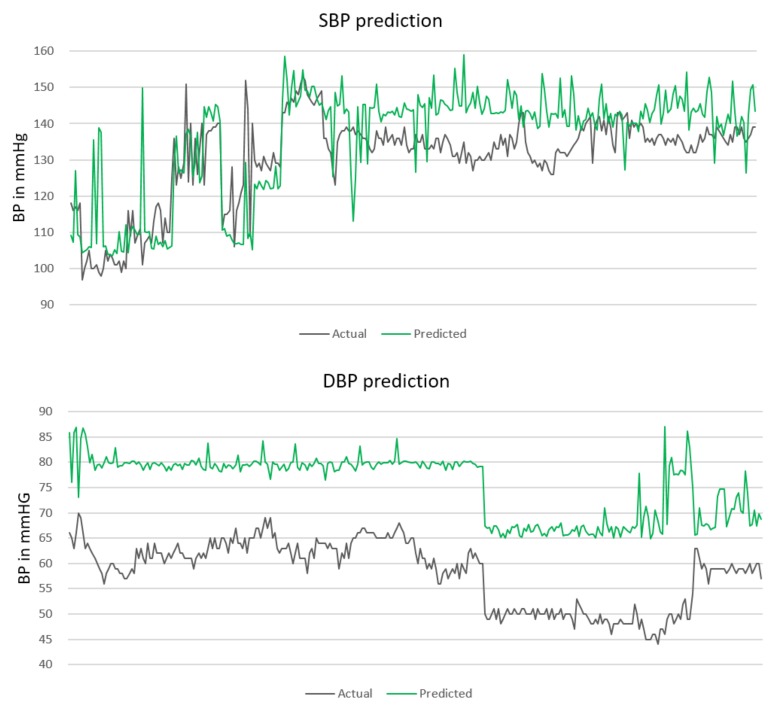
SBP and DBP prediction for testing set.

The absolute blood pressure values for the SBP and DBP prediction are depicted in Figure 2. The actual BP values are marked with a black line. The green line represents the predicted BP values. All BP values are given in mmHg. It can be concluded that the models tend to predict higher BP values than the actual values, especially when predicting the DBP; however, the prediction line still follows the dynamic of the actual BP values.

To estimate the sensitivity of the results, we used the validation set to investigate its influence in the choice of the hyperparameters. We fed the classifier and the regression models one subject from the validation set in each iteration, obtaining the best hyperparameters for the particular setting. However, no significant changes in the parameters were noticed and the following results in Table 7 were obtained when testing the models with the designated testing set.

### 3.3. Calibration Experiments

For solving the problem of miss-predicting the BP values, a calibration method based on the probability distributions of the MAE values obtained from the validation set in the training phase has been proposed. Each SBP, DBP, and MAP error highlighted a test for probability distribution that produced the best fit probability distributions. Their parameters are as follows. The SBP validation set errors showed to fit best in a Generalized Pareto distribution with k=−0.49, σ=19.06, θ=−14.98; the DBP validation set errors showed to fit best in a Logistic distribution with μ=−6.85, σ=5.04, and for MAP the validation set errors showed to fit best in Generalized Pareto distribution with k=−0.17, σ=9.62, θ=−10.34.

**Figure 3:**
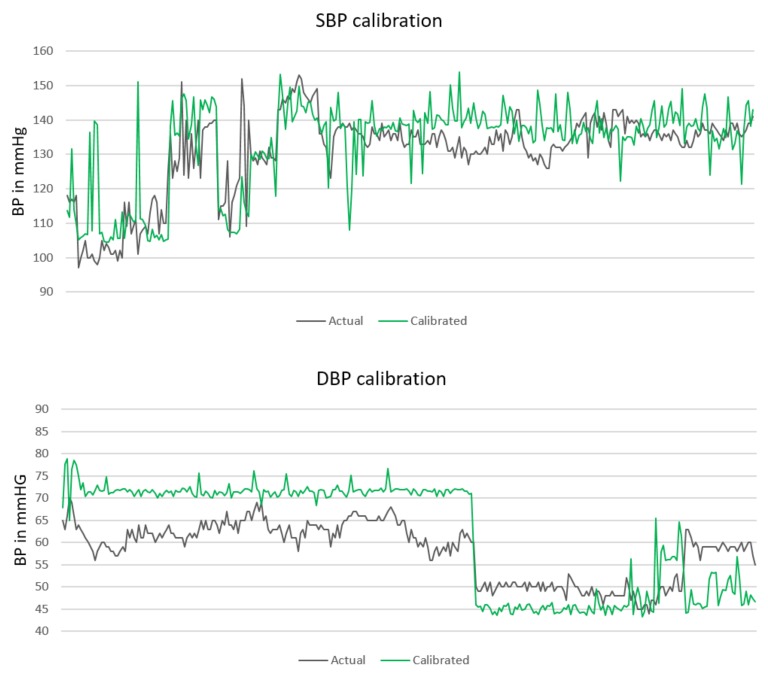
SBP and DBP calibration for testing set.

Having the distributions parameters, for every subject in the testing set, an error from the particular distribution is generated and subtracted from the predicted value. Hereupon, as soon as a new subject is available, a random error from the given probability distribution is generated and is subtracted from the predicted value. Figure 3 presents the calibrated prediction (green line) for the same BP values presented in Figure 2.

The overall MAE and RMSE after the calibration significantly decreases, especially in the DBP case. The new MAE ± SD (RMSE) in mmHg are 7.72 ± 10.22 (10.50) for SBP, 9.45 ± 10.03 (11.07) for DBP, and 8.13 ± 8.84 (10.26) for MAP. The calibration results for each distinct subject are presented in Table 8. Several subjects for which we encountered problems for prediction without calibration, remained problematic in the calibration phase as well. However, a significant improvement was obtained for subjects 14 and 15 (presented in bold in Table 8), which contain most of the instances, 436 and 258, correspondingly. For those subjects, the total error (MAE SBP + MAE DBP + MAE MAP) in Table 8 decreased by 40% in the 14th subject and by 50% in the 15th subject when compared to the total error (MAE SBP + MAE DBP + MAE MAP) in Table 6 for the same subjects.

### 3.4. Feature Analysis

As previously mentioned, we used a band-pass Butterworth filter for keeping the information that is carried between 0.3 Hz and 50 Hz. Choosing the lowest threshold of 0.3 Hz was both experimentally proven and supported by the literature. Considering the fact that valid ECG information is provided even in the range [0.05, 0.5] Hz [76]; to completely remove the baseline, the cut-off frequency must be set higher than the lowest frequency in the ECG [54]. This is necessary in order to prevent some of the baseline to pass as part of the ECG. Therefore, we performed an analysis of how baseline removal at different frequencies affects the performance of the created models. The results are presented in Table 9. Starting from a cut-off frequency of 0.01 up to the threshold of 0.5, where both the baseline and ECG information exist, it can be perceived that the frequency of 0.3 is the point where the models achieve the highest accuracy, since after this threshold the accuracy starts to decrease again.

**Figure 4:**
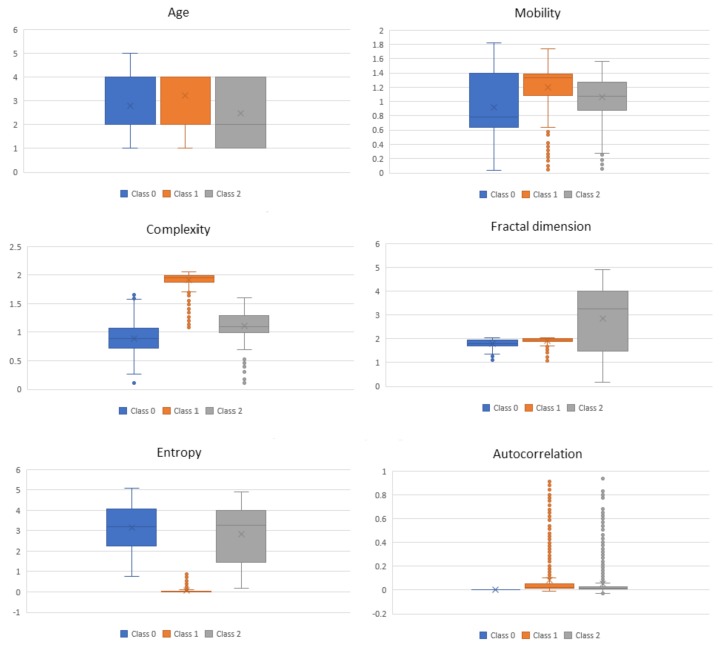
Box-and-whisker plots per class for the complexity features.

For the complexity features obtained from the ECG signals at 0.3–50 Hz, in Figure 4, we present box-and-whisker plots to illustrate the shape of the distributions, the mean value, and the variability of each complexity feature with respect to the three BP classes as described in Table 2. This was done for the purpose of analyzing if a certain feature is distinguishable between the three classes. It can be seen that, for some of the features, e.g., Mobility, Complexity and Entropy, just the mean value itself has a discriminatory power for the three classes. In addition to the mean value, the variability of the feature values also includes some additional information. However, in some cases, the mentioned variability of the feature values may indicate noise in the data.

## 4. Discussion

Our BP estimation system, based on ECG sensor inputs, enabled reliable monitoring of various BP parameters on data obtained from 51 different subjects and four different ECG sensors. The intention of the proposed method is to reveal new insight into the relation between ECG and BP. The relations are represented by the ML models that we determined from the data. We are not aware of any previous study that described this type of relations using this particular choice of features. The datasets we published are to be freely available for scientific purposes [77].

The proposed BP estimation system introduced several novelties which led to a performance close to that of a certified medical device. The first novelty was feature extraction using complexity analysis. Based on the hypothesis that a normal and healthy biomedical system is of high complexity and once an abnormality occurs its complexity drops, these features extracted from the ECG signals seem to contain valuable information regarding BP. The complexity analysis excludes the morphological features of the ECG signals and highlights the entropy of the system, which enables better learning and, consequently, predictions. This was confirmed both by the performance of the overall ML system and later on by the feature analysis, where it can be clearly seen that the distributions of the features change with respect to the different BP classes (Figure 4).

Another novelty that distinguishes our system from the typical “flat” ML approaches for BP estimation is the introduction of the stacking scheme. The stack of several classifiers allows for the meta-learner to receive multiple views over the relations, structures, and patterns in the data, which leads to good performance. The error (MAE) on an unseen testing set is 8.64 for the SBP, 18.20 for the DBP, and 13.52 for the MAP prediction. If a calibration based on validation set errors probability distribution is provided, the MAE significantly decreases to 7.72 for SBP, 9.45 for DBP and 8.13 for MAP. The summary of the results is provided in Table 10. The goal is to achieve results as close as possible to results obtained by what is considered a certified medical device for BP estimation (±5 mmHg, and SD within 8 mmHg according to BHS and AAMI standards [78]).

Considering the time performance of the method, the sensing time needed to acquire ECG signals from the sensor is 30 s; the average time needed for the complexity analysis of the signal is 0.1272 s; the average time needed for the methodology to build the model for prediction is 1.0547 s; and the average time needed for performing a prediction is 0.0001 s. Therefore, once the prediction model is built, the predictions can be considered real-time calculations. From related work, we identified only two other studies in which ECG was used for BP estimation. However, both methods used an additional sensor besides the ECG sensor (i.e., PPG sensor) and achieved errors of ±5.93 (SBP), ±4.76 (DBP), and ±4.23 (MAP) when considering 10 participants [27]; and 7.49 ± 8.8 (SBP) and 4.07 ± 5.6 (DBP) [26]. In contrast to these approaches, our system uses only one sensor and is trained and evaluated on data from 51 participants and four different sensors.

### 4.1. Limitations and Future Work

The method could achieve results close to those achieved by medical device by using probability distributions based calibration. If it is desired to measure BP in other than sedentary conditions, the method should be enriched by an activity recognition module (e.g., by using acceleration sensors [79]). Moreover, a context-based BP estimation may be developed in the future [80]. The method was based and evaluated on all suitable data that we found to be available for this kind of research together with our own developed database with 51 different subjects and four different ECG sensors. However, for robust testing, a larger study with a few hundred diverse participants will be considered.

### 4.2. Comparison with Prior Work

Considering the published results, the achieved mean error for the systolic BP (SBP) and diastolic BP (DBP) estimation is 5.1 ± 4.3 mmHg, and 4.6 ± 4.3 mmHg, respectively, in a case study that encompasses 78 PPG records and matching SBP and DBP values [81]; an error of ±4.76 mmHg for DBP, ±4.23 mmHg for the mean arterial pressure (MAP), and ±5.93 mmHg for SBP is achieved in a pilot study of 150 recordings from 10 subjects [27]; 9 ± 5.6 mmHg for SBP and 1.8 ± 1.3 mmHg for DBP is obtained from a method that uses Ballistocardiography (BCG) and PPG signals [82]; 0.8 ± 7 mmHg for SBP and 0.9 ± 6 mmHg for DBP by using PPG signals [83]; accuracy results of 7.487 ± 8.824 mmHg (mean ± SD) for SBP and 4.076 ± 5.617 mmHg (mean ± SD) for DBP from a PTT-based study [26]; and a similar PTT-based method tested on 300 datasets from six subjects provides a SD of 6.492 mmHg [84]. Table 11 presents a comparison of the results reported in this paper with the results reported in the literature. All results present the MAE ± SD. Our study uses least amount of sensors (one), analyzed data from 51 subjects with the widest age range (16–83), used complexity analysis with a stack of ML, and achieved comparable results to the rest of the studies.

## 5. Conclusions

Our method estimates systolic BP (SBP), diastolic BP (DBP), and the mean arterial pressure (MAP) from ECG sensor data. The predictions are either in the form of three BP classes or in the form of a numeric value representing the absolute BP values. We introduced two novelties: complexity analysis for feature extraction, and stack of ML models for more robust predictive models. When probability distribution based calibration is provided, the results are close to those of a certified medical device. The first contribution of this study is the establishment of a design relationship between BP and ECG in the form of ML models. The second contribution is of practical value—the user with an ECG sensor needs no additional device for measuring BP. Since the trend of ECG sensor usage indicates a continuous increase in demand, we believe that our proposed solution has promising real-world applications in civilian and military environments.

## Figures and Tables

**Table 1:** Datasets summary information.

Dataset	Reliability	Number of Participants	Age	Status
Cooking hacks sensor [38]	[39,40,41]	16	16–72	healthy
180 eMotion FAROS [42]	[43,44,45]	3	25–27	healthy
Zephyr Bioharness module [46]	[47,48,49,50]	25	20–73	14 healthy, 11 unhealthy
Charis Physionet database [51]	Clinical equipment	7	20–74	brain injuries

**Table 2:** Rules and categorization.

Category		SBP (mmHg)	Logical	DBP (mmHg)	Number of Instances	Grouped
Normal	**HPTN**	≦90	OR	≦60	25	312
	**N**	90–119	AND	60–79	287		
Prehypertension	**PHTN**	120–139	OR	80–89	1091	1091
Hypertension	**S1HTN**	140–159	OR	90–99	83	1726
	**S2HTN**	≧160	OR	≧100	12		
	**ISHTN**	≧140	AND	<90	1605		
	**HTNC**	≧180	OR	≧110	26		

**Table 3:** Number of instances per dataset.

Sensor/Class	0	1	2
1	197	85	15
2	4	25	6
3	44	28	24
4	67	953	1681
Total	312	1091	1726

**Table 4:** Age mapping rules and number of instances in each group.

Category	Age Range	Number of Instances (Samples)
Adolescence	12–20	845
Early Adulthood	21–35	643
Midlife	36–50	59
Mature Adulthood	51–80	1581
Late Adulthood	>80	1

**Table 5:** Classification and regression models evaluation on validation set.

Accuracy (%)	Kappa	MAE SBP	MAE DBP	MAE MAP	RMSE SBP	RMSE DBP	RMSE MAP	Corr.
73.04	0.40	5.39	7.01	5.08	7.17	8.16	6.50	0.39
89.80	0.40	6.51	4.68	5.32	7.93	5.97	6.57	0.35
76.79	0.55	9.47	7.54	7.93	14.68	11.47	12.10	0.44
91.76	0.76	5.17	7.99	5.20	7.82	8.98	6.47	0.27
78.05	0.60	13.27	9.21	4.59	17.24	11.77	6.43	0.77
**85.71**	**0.78**	**7.86**	**9.62**	**6.00**	**9.46**	**11.19**	**7.44**	**0.77**
87.50	0.76	7.38	8.47	7.69	9.72	10.57	9.60	0.87
76.00	0.58	10.13	6.38	7.97	13.04	8.65	9.86	0.35

**Table 6:** MAE and RMSE evaluation for SBP, DBP and MAP.

Patient	Number of Instances	MAE SBP	RMSE SBP	MAE DBP	RMSE DBP	MAE MAP	RMSE MAP
1	5	8.69	8.77	3.80	4.86	5.66	6.21
2	20	9.48	15.67	4.74	6.93	6.73	11.21
3	10	7.77	16.06	4.22	7.98	5.39	10.58
4	11	7.15	8.56	9.87	10.69	7.71	8.48
5	12	8.00	9.52	11.51	12.08	8.31	8.92
6	8	6.42	8.84	7.42	8.64	5.27	7.23
7	5	7.46	10.03	12.56	13.00	6.92	7.21
8	5	10.33	11.63	5.10	5.82	3.02	3.68
9	1	22.60	22.60	11.05	11.05	14.27	14.27
10	1	35.67	35.67	12.87	12.87	20.87	20.87
11	1	1.00	1.00	6.36	6.36	1.98	1.98
12	1	34.85	34.85	23.02	23.02	27.31	27.31
13	12	5.66	6.10	6.90	9.15	7.12	8.10
14	436	8.48	10.36	19.56	20.09	16.67	17.38
15	258	8.94	11.28	19.74	20.63	10.54	12.21

**Table 7:** Models hyperparameters testing.

MAE SBP	RMSE SBP	MAE DBP	RMSE DBP	MAE MAP	RMSE MAP
8.18	10.79	17.44	18.70	13.98	15.66
9.76	12.81	17.06	18.39	11.66	13.38
11.90	15.33	16.83	18.16	11.41	13.55
9.35	12.03	17.76	19.04	12.42	14.16
10.54	13.48	17.01	18.27	10.52	12.36

**Table 8:** Calibrated MAE and RMSE evaluation for SBP, DBP and MAP.

Patient	Number of Instances	MAE SBP	RMSE SBP	MAE DBP	RMSE DBP	MAE MAP	RMSE MAP
1	5	6.69	8.04	5.84	7.95	10.08	11.57
2	20	10.34	16.23	9.64	10.62	5.72	10.02
3	10	8.01	16.37	13.33	13.55	8.18	13.66
4	11	7.47	8.99	26.71	27.35	31.33	32.31
5	12	10.83	13.20	4.17	5.15	4.49	5.23
6	8	7.92	9.86	13.61	15.00	6.38	7.09
7	5	5.96	9.17	5.16	7.94	4.90	7.02
8	5	9.89	11.02	7.04	7.44	22.14	22.43
9	1	28.55	28.55	12.09	12.09	14.58	14.58
10	1	27.98	27.98	17.56	17.56	40.04	40.04
11	1	4.24	4.24	2.17	2.17	0.54	0.54
12	1	28.18	28.18	31.25	31.25	36.43	36.43
13	12	2.74	3.83	15.13	16.27	16.19	16.62
**14**	**436**	**6.90**	**8.64**	**11.45**	**12.31**	**8.50**	**9.55**
**15**	**258**	**8.75**	**12.23**	**5.13**	**6.34**	**6.06**	**8.14**

**Table 9:** Complexity features performance for different cut-off frequencies.

Cut-off Frequency	MAE SBP	RMSE SBP	MAE DBP	RMSE DBP	MAE MAP	RMSE MAP	Mean MAE	Mean RMSE
0.01	28.77	31.13	17.03	19.06	13.42	18.30	19.74	22.83
0.03	25.36	28.33	17.36	19.37	14.27	18.51	19.00	22.07
0.05	24.70	27.66	18.20	20.18	14.07	18.40	18.99	22.08
0.10	11.30	14.90	17.56	18.86	12.39	14.42	13.75	16.06
**0.30**	**8.64**	**10.97**	**18.20**	**19.34**	**13.52**	**15.07**	**13.46**	**15.13**
0.50	8.82	11.38	18.18	19.28	15.47	17.31	14.16	15.99

**Table 10:** Results summary.

Error (mmHg)	Prediction	Calibration
MAE SBP	8.64 ± 10.74	7.72 ± 10.22
RMSE SBP	10.97	10.50
MAE DBP	18.20 ± 8.45	9.45 ± 10.03
RMSE DBP	19.34	11.07
MAE MAP	13.52 ± 8.06	8.13 ± 8.84
RMSE MAP	15.07	10.26

**Table 11:** Comparison results with prior work.

Study	Source	Number of Subjects	Age	Records	Method	MAE SBP	MAE DBP	MAE MAP
[81]	PPG	65	22–65	78	Wavelet, SVM	5.1 ± 4.3	4.6 ± 4.3	N/A
[27]	ECG, PTT-CP	10	24–63	150	Numerical solution	±5.93	±4.76	±4.23
[82]	BCG, ECG	5	/	/	Analytical solution	9 ± 5.6	1.8 ± 1.3	N/A
[83]	PPG	16	18–48	/	Frequency analysis	0.8 ± 7	0.9 ± 6	N/A
[26]	ECG, PPG, PPT	/	/	/	Analytical solution	7.49 ± 8.8	4.07 ± 5.6	N/A
[85]	PPG	MIMIC II [86]	adults	4254	Linear Regression, ANN, SVM	13.84 ± 17.56	6.96 ± 9.16	8.54 ± 10.87
[87]	PTT	127	/	/	Wavelet transforms	±7.63	N/A	N/A
[88]	PTT, PPG	27	21–29	/	Analytical solution	−0.37 ± 5.21	−0.08 ± 4.06	−0.18 ± 4.13
Our results	ECG	51	16 – 83	3129	Complexity analysis + ML	7.72 ± 10.22	9.45 ± 10.03	8.13 ± 8.84

## Abbreviations

The following abbreviations are used in this manuscript:

BCG	Ballistocardiography
BP	Blood pressure
DBP	Diastolic blood pressure
ECG	Electrocardiogram
EU	European Union
HPTN	Hypotension
HTNC	Hypertensive crisis
ISHTN	Isolated systolic hypertension
LODO	Leave one dataset out
LOSO	Leave one subject out
MAE	Mean absolute error
MAP	Mean arterial pressure
ML	Machine Learning
N	Normal
PAT	Pulse Arrival Time
PHTN	Prehypertension
PPG	Photoplethysmogram
PTT	Pulse transit time
PWV	Puse wave velocity
RMSE	Mean squared error
S1HTN	Stage 1 hypertension
S2HTN	Stage 2 hypertension
SBP	Systolic blood pressure
SD	Standard deviation
WHO	World Health Organization
